# Development of a Novel Dietary Assessment Method Using Gamification Concepts: Exploratory and Application Study

**DOI:** 10.2196/72387

**Published:** 2026-03-13

**Authors:** Yulin Zhang, Tianwu Lin, Yuefeng Tan, Xiaojin Shi, Yucheng Yang, Haibing Yang, Xiaona Na, Junhan Zhang, Ai Zhao

**Affiliations:** 1 Vanke School of Public Health, Tsinghua University, Jinchunyuan Building, Tsinghua University, 30th Shuang Qing Road, Hadian District, Beijing, 100084, China, 86 13811131994; 2 Shenzhen Yantian Maternal and Child Health Hospital, Shenzhen, Guangdong, China

**Keywords:** dietary assessment, gamification, dietary intake, food preference, eating behavior tendencies

## Abstract

**Background:**

Childhood and adolescent malnutrition, encompassing undernutrition and overnutrition, poses significant global health challenges, necessitating comprehensive dietary assessment tools. Existing dietary assessment methods, such as 24-hour dietary recalls (24HR), often fail to capture eating behaviors and food preferences.

**Objective:**

This study aims to compare a newly developed gamified dietary assessment tool (GDA) with a traditional 24HR and to explore its applicability in assessing dietary behavior tendencies among children and adolescents.

**Methods:**

A 2-phase study was designed, including an exploratory and an application study. The exploratory study included 30 school-aged participants, comparing the GDA with the 3-day 24HR. Nutrient and food intakes were analyzed using Pearson or Spearman correlation coefficients and Bland-Altman plots. The application study, conducted among 1541 adolescents (11‐18 y), assessed dietary intake and eating behavior tendencies. Differences in dietary intake across age, gender, socioeconomic status, and weight status were analyzed using the Kruskal-Wallis rank sum test. Multiple linear regression models were used to examine the associations of dietary intakes with emotional eating and with dining environment tendencies, respectively.

**Results:**

In the exploratory study (n=30), the GDA demonstrated moderate agreement with 24HR for energy (*r*=0.46, *P*=.01) and carbohydrates (*r*=0.50, *P*=.005). Bland-Altman plots indicated good agreement for energy and carbohydrate intake between methods (mean differences around 0). For fat intake, although the mean difference was close to 0, the correlation was not statistically significant. In contrast, the GDA substantially overestimated protein intake (mean difference around 25 g). In the application study (n=1541), higher emotional eating scores were associated with higher snack consumption (*β*=0.438, 95% CI 0.035-0.840), and with lower protein (*β*=−0.159, 95% CI −0.267 to −0.052), fruit (*β*=−0.464, 95% CI −0.854 to −0.073), and nut consumption (*β*=−0.183, 95% CI −0.304 to −0.062). Participants who chose solitary screen eating consumed significantly more carbohydrates than those who selected “eat with peers” (*β*=4.2, 95% CI 1.2-7.1).

**Conclusions:**

This study demonstrates that the GDA effectively captures both dietary intake and contextual eating behaviors in young populations, providing data distinct from traditional methods, such as 24HR. As a complementary assessment approach, it offers valuable insights into food preferences and eating patterns through its interactive design.

## Introduction

Children and adolescents have been facing a growing burden of malnutrition worldwide [1]. It is estimated that more than 390 million children and adolescents around the world are overweight, of whom 41% are obese [2]. At the same time, in low- and middle-income countries, approximately 16% of adolescents experience stunting and thinness [3], while micronutrient deficiencies have increasingly become a major threat to their development [4]. Malnutrition during childhood and adolescence can lead to substantial long-term health consequences in adulthood. Children in the highest BMI quartile could have more than twice the rate of death from endogenous causes compared to those in the lowest quartile [5], and obesity in adolescence also predicts a higher risk of type 2 diabetes [6] and cardiovascular disease [7] in adulthood. Also, early undernutrition has long-lasting effects on energy expenditure, lipid metabolism, insulin resistance, and mental health, which may even be passed to the next generation [8]. Addressing these issues requires a better understanding of dietary patterns and the factors that influence food choices among children and adolescents. Traditional and emerging dietary assessment methods are both utilized to evaluate nutritional intake. Traditional methods, including retrospective methods (eg, 24-hour dietary recalls [24HR]) and prospective methods (eg, weighed food records), have long been used in research and clinical practice to capture individuals’ food consumption [9]. Meanwhile, emerging technologies, including mobile apps [10], wearable devices [11], and virtual reality [12,13], are increasingly used to assist in dietary surveys. For retrospective methods, the main challenge is their reliance on self-reporting, which can be prone to bias, especially in younger populations. Young children have limited ability to recall, estimate, and cooperate in dietary investigations [14], and their diets and eating habits tend to be more variable compared to those of adults [15]. Prospective methods are often challenging to implement due to high labor costs and also rely on respondents’ self-reports. Their food choices may be influenced by their tendency to report socially desirable food consumption [16], while novel methods, such as mobile apps, often underestimate the intake of energy or specific nutrients [10,17]. Additionally, all these methods were unable to capture the broader contextual dietary factors, such as eating environment and food preferences, that influence dietary behaviors in adolescents. Particularly, children’s diets are typically shaped by their families or school environment [18], which may obscure their food preferences, including adverse tendencies toward unhealthy eating habits. It is reported that children would select different foods when they were away from their parents compared to when they were watched [19]. When food is freely available and parents are absent, they always tend to choose more restricted foods, often considered unhealthy, over unrestricted options, which may contribute more to their health and development [20].

To address these limitations, a combination of gamification and dietary assessment could be a potential solution. Gamification, defined as the use of game design elements in nongame contexts [21], is an emerging interactive digital strategy that has gained widespread application and research across various fields [22,23]. In the domains of health, gamification primarily targets children for chronic disease management [24], physical activity [25], mental health [26], and nutrition education [27,28]. To the best of our knowledge, there is a lack of studies applying gamification in dietary surveys and assessments, especially for children and adolescents.

Therefore, we developed a new gamified dietary assessment tool (GDA) that engages children and adolescents in a simulated autonomous eating scenario, aiming to capture their authentic food preferences and eating behavior tendencies. An exploratory comparison study was conducted to compare the GDA with 24HR, followed by an application study to explore how the GDA captures dietary behavior tendencies beyond the scope of traditional assessment methods. Through the integration of gamification, we seek to determine whether this innovative method can provide a more comprehensive understanding of young individuals’ nutrition status and true dietary tendencies.

## Methods

### Study Design and Population

The study comprised 2 parts. The first is an exploratory study, aiming to compare the hypothetical food choices assessed by the GDA with the actual dietary intake estimated through a traditional 24HR. The second part is an application study, using the GDA to simulate the eating process of children and adolescents to assess their dietary intake and tendencies of eating behaviors.

The exploratory study was a cross-sectional study assessing the agreement of the interviewer-led 3-day 24HR and the GDA. The research took place at an elementary school in Changzhou, Jiangsu province, China. A total of 30 school-aged children were recruited voluntarily. The exclusion criteria for participants were (1) patients with metabolic diseases or other conditions requiring a special diet and (2) patients with eating disorders or other disorders that significantly impact eating behaviors.

The application study was conducted among school-aged children and adolescents at a middle school in Lanzhou, Gansu province, China. Cluster sampling was used to select 40 classes, which contained 2241 students. The inclusion and exclusion criteria were the same as the exploratory study. To ensure data quality, we further excluded (1) the ones with abnormal energy intake (energy intake exceeding 4000 kcal or below 500 kcal), (2) participants who did not complete the Emotional Eating Scale, and (3) those who failed the attention check questions.

### Ethical Considerations

The study was approved by the ethics committee of Institutional Review Board of Tsinghua University (Project No: 20220102). All participants and their parents were required to give consent before participating in the study, through which they were informed about the procedures and other related information of the study. No compensation was provided to participants. All human participant data have been anonymized or deidentified to protect participants’ privacy, with no personal identifiers retained in records or analyses.

### Data Collection

Data collection for 24HR and GDA in the exploratory study took place in May 2023. The first 24HR interview was conducted on day 1 by trained researchers, followed by the self-administered GDA survey on the same day. The remaining 2 24HR interviews were conducted on days 2 and 3, respectively.

In the application study, basic information was collected by trained researchers using a standard protocol. BMI-for-age *z* score (BMI *z* score) was then calculated according to the growth reference standards for children and adolescents aged 5 to 19 years provided by the World Health Organization [29]. The severity of emotional eating behaviors was assessed by emotional eating scores (EESs) based on the Emotional Eating Scale [30]. This scale is based on 1 dimension of the Intuitive Eating Scale-2, specifically the “Eating for Physical Rather than Emotional Reasons” dimension, which includes 8 items, 4 of which are reverse-scored. As no version specifically validated for Chinese children and adolescents is currently available, a widely applied general version of the EES was adopted. Participants were instructed, “For each item, please check the answer that best characterizes your eating attitudes or behaviors.” The item-response scale was a 5-point scale, where 1 indicates strongly disagree, 2 indicates disagree, 3 indicates neutral, 4 indicates agree, and 5 indicates strongly agree. Reverse-scored items were rated inversely, with 1 indicating strongly agree to 5 indicating strongly disagree. EES ranged from 5 to 40, with higher scores indicating a greater likelihood of engaging in emotional eating behaviors as a way to cope with negative emotions. The EES was self-administered by participants via the digital platform under the supervision and guidance of trained investigators. Dietary intake data and dietary behavior data were collected with the GDA.

### Gamified Dietary Assessment Tool

**Figure 1.**
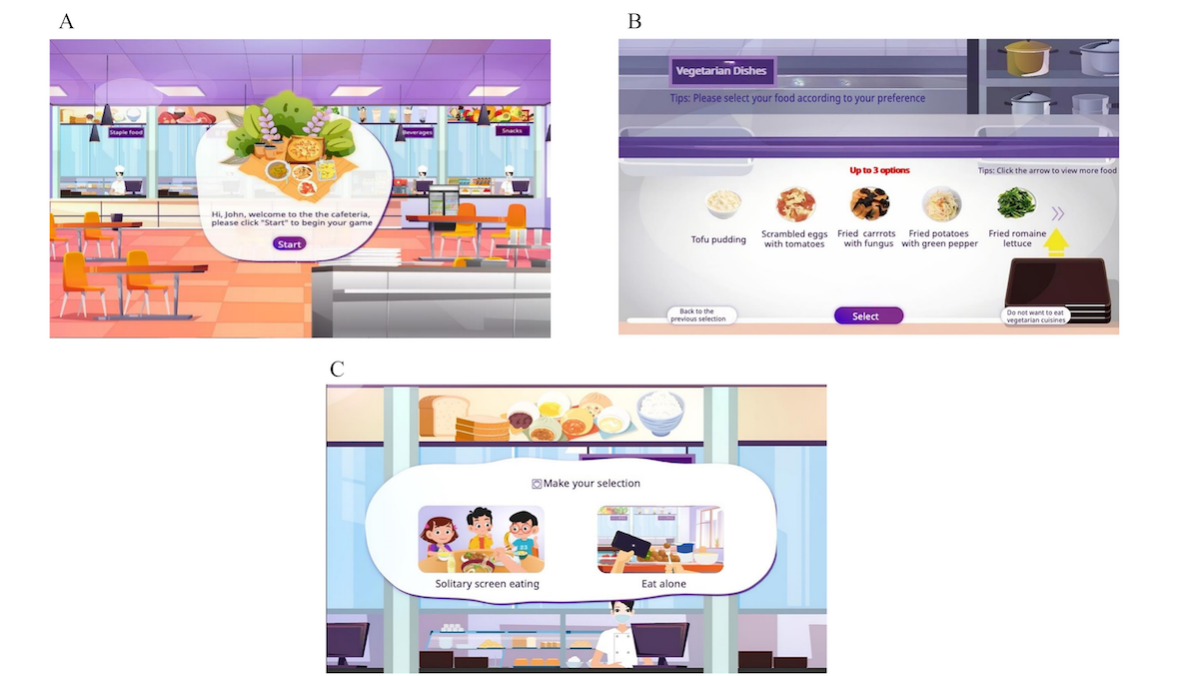
Three primary modules designed in the gamified dietary assessment tool (GDA): (A) module of guided tutorial, (B) module of food selection, and (C) module of dining scenario selection.

The dietary assessment tool was designed with gamification principles to improve user engagement and accuracy. Core gamification elements included (1) a drag-and-drop interaction mechanic for food selection, (2) a dynamic progress bar and encouraging prompts for real-time feedback, and (3) a visually engaging interface with animations and child-friendly graphics to reduce cognitive burden and enhance immersion. These features aimed to transform traditional dietary recall into a more intuitive and motivating experience for children and adolescents. The GDA consisted of 3 primary modules (Figure 1A-C): the guided tutorial module, the food selection module, and the dining scenario selection module. In the guided tutorial module, the GDA offered prompts to instruct participants on how to complete the survey. Participants then proceeded to the second module to choose foods for breakfast, lunch, and dinner in sequence under a virtual buffet situation. Children were asked to make their independent decisions on what types of dishes or beverages and what quantities they wished to consume by dragging and dropping food portions from the buffet table. There was a total of 66 dishes or beverages, which consisted of 81 food items belonging to 10 main food groups, including cereals, tubers, vegetables, fruits, livestock and poultry, aquatic products, eggs, dairy products, legumes, nuts, as well as 2 groups of processed food (including snacks and beverages). Specifically, alcoholic beverages were included in the GDA food list to reflect real-life dietary exposure and to allow the identification of potentially harmful alcohol intake tendencies, although alcohol intake data were not analyzed in this study. Dishes were designed by nutritional experts according to the dietary culture of China and their nutritional contents. The details of dish and beverage selection are shown in Multimedia Appendix 1. After completing the food selection, participants were supposed to select the dining scenarios, with 2 options available: “solitary screen eating” and “eat with peers.” Participants who chose “eat with peers” were presented with an animation featuring several children engaging in cheerful conversation while eating. In contrast, those who selected “solitary screen eating” were presented with an animation depicting a child eating while watching cartoons on a mobile phone. The main outputs of the GDA encompassed eating behavior tendencies, estimated food intake, and nutrient intake based on the selected food items in the GDA.

### Food, Energy, and Nutrient Intake Assessment

In an exploratory study, the quantitative measurement of food intake by 24HR was assisted by Photographic Atlas of Food Portions for Accurate Quantification of Dietary Intakes in China [31] and standard utensil models, which allowed participants to estimate portion sizes by comparing their intake against standardized images and models. Thereafter, energy and nutrient intake were calculated according to the China Food Composition Tables, Sixth Standard Edition [32]. The 3-day average intakes of energy and nutrients were used in statistical analysis.

For the GDA, all mixed dishes were decomposed into their constituent ingredients. Energy and nutrient intakes in both exploratory and application studies were calculated by summing the nutrient values of each component based on China Food Composition Tables, Sixth Standard Edition [32].

### Data Analysis

The Shapiro-Wilk test for normality indicated that the energy intake and the nutrient intake in the exploratory study were not normally distributed. Consequently, descriptive statistics for energy and nutrient intake were reported as medians with interquartile ranges. The logarithmic transformation of the variables was performed, as dietary data on energy, nutrient, and food intake typically exhibit a right-skewed distribution that can be effectively normalized by this transformation, and Pearson correlation analysis was used to examine the association between the 2 dietary assessment methods regarding these intake measures. If the log-transformed data still did not follow a normal distribution, Spearman rank correlation analysis was then used to assess the correlation between the 2 methods. Additionally, Bland-Altman plots were constructed to visually assess the agreement between the 24HR and the GDA. This method involves plotting the mean value of the 2 measurements on the x-axis and the difference between them on the y-axis, with the mean difference and 95% limits of agreement displayed to evaluate the consistency between the 2 methods.

In the application study, descriptive statistics for energy intake, nutrient intake, food intake, and macronutrient energy ratio were also presented as medians with interquartile ranges. A comparison of demographic characteristics was also conducted between the included population and those who failed the attention check question using the Wilcoxon test and Fisher exact test. The distribution of macronutrient energy ratios was described and compared with the Dietary Reference Intakes for China [33]. The Kruskal-Wallis rank sum test was used to compare the energy intake, nutrient intake, food intake, and macronutrient energy ratio among participants with different sociodemographic characteristics, while Fisher exact test was used to compare the distributions of macronutrient energy ratio across different categorical groups due to small numbers in certain distribution groups. We used multiple linear regression models to examine the association between EES and nutritional outcomes. Age, sex, and socioeconomic status were included as covariates. Energy intake was divided by 10 due to the large values of energy intake. Stratified analysis was further conducted according to BMI *z* scores. BMI *z* scores less than –2 were defined as “thin,” scores −2 or higher and 1 or less as “normal,” and scores greater than 1 as “overweight and obese” [34]. The ß-values and 95% CI for each model were calculated to assess the effects of emotional eating behavior tendencies on nutritional outcomes. Three models were applied: model 1 made no adjustments; model 2 adjusted for age, sex, and socioeconomic status; and model 3 further adjusted for energy intake. Additionally, dynamic scatter plots were generated using the “gganimate” package in R, illustrating the association between EES and macronutrient intake across different BMI *z* score groups to visualize how emotional eating affects energy and macronutrient intake across weight statuses. Multiple linear regression models were also used to evaluate the association between dining environment tendencies and nutritional outcomes.

Statistical analysis was conducted using the R software (version 4.3.0; Posit PBC), with all tests being 2-sided and a significance level set at .05.

## Results

### Exploratory Study

A total of 30 school-aged children were recruited, including 9 (30%) male participants and 21 (70%) female participants. Participants ranged in age from 9 to 11 years, with an average age of 10.7 years (SD 0.55). Nutrient data from the GDA and 24HR were not normally distributed. Consequently, a logarithmic transformation was applied to the energy and nutrient intake values. Pearson correlation analysis of the transformed data showed significant positive correlations between the 2 methods for energy intake, carbohydrates, cholesterol, and a few micronutrients (vitamin A and vitamin C). No significant correlations were observed for the other nutrients, as presented in Table 1.

**Table 1.** Correlation between intakes of energy, nutrients, and main food groups in 24-hour dietary recalls (24HR) and the gamified dietary assessment tool (GDA).

Variables	GDA, median (IQR)	24HR, median (IQR)	Correlation coefficient	*P* value
Energy and nutrients				
Energy (kcal)	1596.8 (1370.3-1895.3)	1510.8 (1335.0-1841.8)	0.46	.01
Protein (g)	81.4 (63.3-94.0)	48.7 (42.4-57.0)	0.28	.13
Fat (g)	73.0 (46.9-87.4)	67.8 (58.0-80.5)	0.31	.10
Carbohydrate (g)	170.5 (129.3-209.1)	188.1 (167.8-220.5)	0.50	.005
Cholesterol (mg)	624.0 (392.4-746.3)	328.9 (219.3-448.6)	0.38	.04
VA (µgRAE)	377.2 (224.7-525.3)	343.9 (259.7-486.5)	0.41	.03
VB1 (mg)	0.5 (0.3-0.6)	0.6 (0.4-0.7)	−0.17	.37
VB2 (mg)	1.0 (0.8-1.2)	0.6 (0.5-0.7)	0.23	.21
Niacin (mg)	15.4 (9.9-20.7)	9.4 (6.9-10.8)	−0.02	.91
VC (mg)	63.5 (41.2-84.0)	30.3 (41.5-54.7)	0.52	.003
VD (µg)^a^	3.3 (2.4-4.3)	0 (0-0)	0.03	.86
VE (mg)	13.2 (11.5-16.8)	30.8 (25.8-37.4)	0.01	.94
Ca (mg)	525.0 (396.0-631.2)	461.7 (327.6-561.9)	0.07	.71
P (mg)	977.7 (756.0-1134.1)	684.7 (538.5-782.3)	0.01	.95
K (mg)	1987.2 (1585.8-2255.6)	1287.3 (1032.5-1740.6)	0.31	.10
Na (mg)	2446.9 (1797.3-3061.4)	5054.7 (4541.2-5710.1)	0.07	.72
Mg (mg)	241.0 (189.8-269.4)	193.2 (152.4-243.9)	0.24	.19
Fe (mg)	15.2 (12.0-17.9)	11.3 (9.1-16.1)	−0.01	.96
Zn (mg)	10.4 (7.6-61.5)	6.7 (6.0-8.3)	0.26	.16
Se (µg)	51.8 (35.3-76.9)	25.3 (20.4-32.5)	0.36	.05
Cu (mg)	1.3 (0.9-9.5)	1.0 (0.8-1.3)	0.17	.38
Mn (mg)	2.2 (1.9-2.5)	2.0 (1.5-2.5)	0.08	.69
Main food groups				
Cereals (g)	194.2 (160.0-267.1)	205.0 (168.9-257.0)	0.44	.01
Tubers (g)	58.5 (0-58.5)	6.7 (0-17.5)	0.11	.55
Vegetables (g)	181.1 (129.1-241.1)	73.3 (41.7-125.8)	0.34	.07
Fruits (g)	60.0 (30.6-61.2)	55.5 (0-132.2)	0.41	.02
Livestock and poultry (g)	206.3 (137.6-247.3)	80.8 (53.1-118.7)	0.18	.34
Aquatic products (g)	90.0 (0-150.0)	0 (0-19.2)	0.25	.18
Eggs (g)	41.0 (36.8-73.8)	37.5 (20.0-60.0)	0.15	.43
Dairy products (g)	124.5 (55.4-176.7)	141.7 (83.3-226.7)	0.53	.003
Legumes (g)	0.0 (0-58.4)	0 (0, 7.5)	−0.28	.13
Nuts (g)^a^	0.0 (0.0-30.0)	0.0 (0.0-0.0)	−0.26	.17

a The intake of vitamin D and nuts showed minimal variability in 24-hour dietary recalls, which limits the interpretability of correlation coefficients for these variables.

Food intake data were nonnormally distributed, requiring logarithmic transformation, but the transformed data still remained nonnormally distributed. Thus, Spearman rank correlation analysis was conducted. Significant positive correlations were observed between 24HR and the GDA for the intake of cereals, fruits, and dairy products, while no significant correlations were found for other food categories (Table 1).

**Figure 2.**
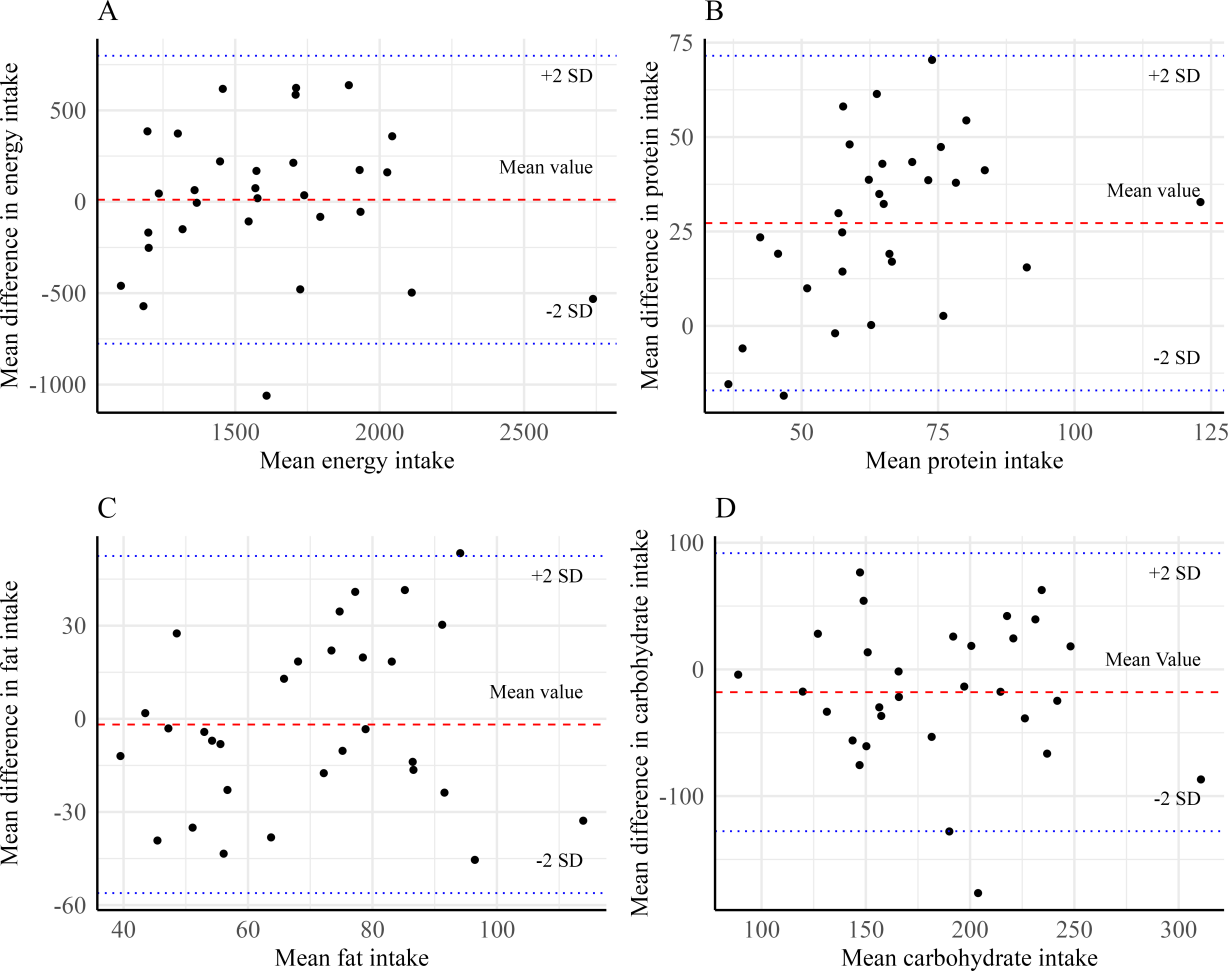
Bland-Altman plots comparing the intake of energy and macronutrients in 24-hour dietary recall (24HR).

The Bland-Altman plot showed that the mean differences in energy (Figure 2A), fat (Figure 2C), and carbohydrate (Figure 2D) intake between the 24HR and the GDA were centered around zero, with all observations evenly distributed within the 95% CI on both sides of the mean difference, which indicated good agreement between the 2 methods. However, the mean difference for protein intake (Figure 2B) was approximately 25 g, suggesting that the GDA may indicate a higher protein intake compared with 24HR.

### Application Study

**Figure 3.**
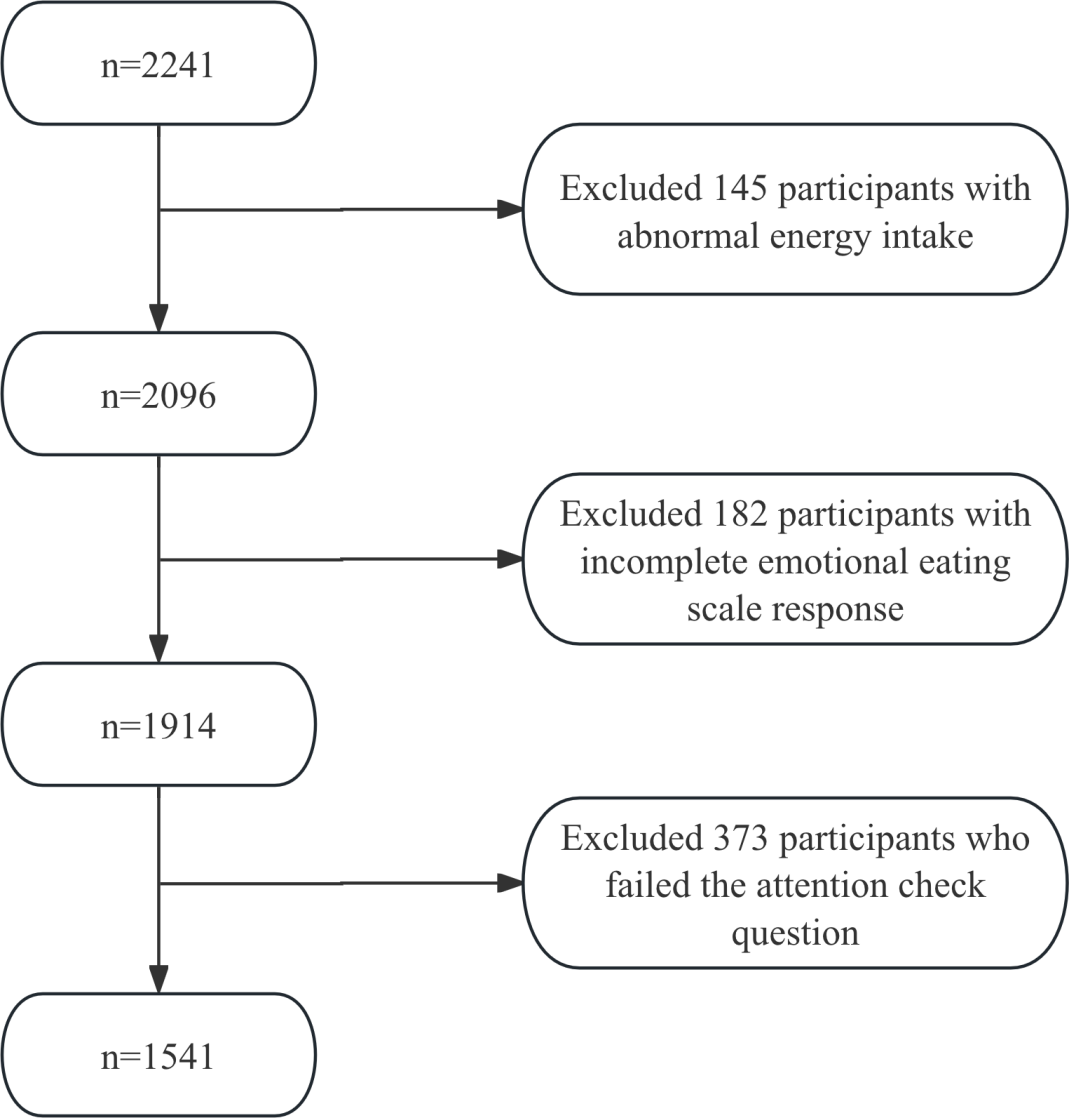
Flowchart of the application study.

In the application study, a total of 2241 participants were initially recruited, resulting in 1541 valid samples (Figure 3), comprising 949 (61.6%) male participants and 592 (38.4%) female participants with an age range of 11 to 18 years and a mean age of 15.2 (SD 1.6) years. Participants excluded due to failed attention checks were significantly younger than those included in the final sample (*P*<.001).

The median GDA-estimated energy, protein, fat, and carbohydrate intakes were 1877.3 (IQR 1410.0-2221.8) kcal, 96.4 (IQR 69.5-113.9) g, 74.0 (IQR 52.5-96.8) g, and 202.0 (IQR 156.3-238.7) g, respectively. Participants aged 11 to 14 years showed a significantly greater tendency to intake more carbohydrate compared to those aged 15 to 18 years (*P*<.001). Male participants demonstrated a higher tendency to intake more energy (*P*=.003), carbohydrate (*P*=.04), protein (*P*=.01), and fat (*P*=.01) than female participants. However, no significant differences were observed in GDA-estimated energy and macronutrient intake across different weight status levels or socioeconomic status levels. The median GDA-estimated intake of various food groups among the participants was 210.1 (IQR 159.7-270.0) g for cereals, 58.5 (IQR 0-58.5) g for tubers, 201.0 (IQR 126.7-279.3) g for vegetables, 30.6 (IQR 0-61.2) g for fruits, 250.5 (IQR 156.2-340.0) g for livestock and poultry, 170.0 (IQR 90.0-250.0) g for aquatic products, 39.0 (IQR 12.9-67.1) g for eggs, 45.7 (IQR 11.1-148.1) g for dairy products, 0 (IQR 0-58.4) g for legumes, and 0 (IQR 0-0) g for nuts, 240.0 (IQR 120.0-360.0) mL for beverages, and 78.0 (IQR 48.0-106.8) g for snacks. Significant differences were found in GDA-estimated intake of cereals (*P=*.02), fruits (*P=*.003), and legumes (*P=*.02) across age groups. Sex differences were also observed, with male participants tending to consume significantly more cereals (*P<*.001), vegetables (*P<*.001), livestock and poultry (*P=*.02), and legumes (*P=*.009) than female participants, while female participants had a higher GDA-estimated intake of fruits (*P<*.001) and dairy products (*P<*.001) than male participants. No significant differences were observed in other food groups across different weight status levels or socioeconomic status levels (Multimedia Appendix 2).

The median ratios of GDA-estimated protein energy contribution, fat energy contribution, and carbohydrate energy contribution were 20.4% (IQR 18.4%-22.1%), 35.8% (IQR 31.4%-41.1%), and 44.0% (IQR 39.7%-49.0%), respectively. The 11- to 14-year group had a higher carbohydrate energy ratio (*P<*.001) and a lower fat energy ratio (*P<*.001) compared with the 15- to 18-year group. Sex difference was also observed, with female participants having a higher carbohydrate energy ratio (*P*=.002). A total of 42.9% (n=661) of the participants had a protein energy ratio within the acceptable macronutrient distribution range (AMDR) (10%‐20%). 17.9% (n=276) of the participants had a fat energy ratio within the AMDR (20%‐30%). 20.1% (n=310) of the participants had a carbohydrate energy ratio within the AMDR (50%‐65%). Only the distribution of carbohydrate energy intake ratios (*P=*.02) showed significant differences across gender (Multimedia Appendix 3).

Furthermore, we explored the association between emotional eating behavior and nutritional outcomes among participants using the GDA. The median (IQR) EES was 22.3 (19-25). After adjusting for age, sex, and socioeconomic status, multiple linear regression analysis showed that higher EESs were significantly associated with a greater tendency to select more carbohydrates, while no significant associations were found for protein or fat selections. After further adjusting for energy intake tendencies, higher EESs were associated with a lower protein intake tendency, with no significant associations observed for fat or carbohydrate intake tendencies (Table 2).

**Table 2.** The association between gamified dietary assessment tool (GDA)–estimated intakes of energy, macronutrients, food groups, and emotional eating scores.

Variables	β (95% CI)		
	Model 1^a^	Model 2	Model 3
Energy/10 (kcal)	0.202 (−0.276 to 0.680)	0.348 (−0.136 to 0.832)	—^b^
Protein (g)	−0.052 (−0.307 to 0.202)	0.009 (−0.249 to 0.267)	−0.159 (−0.267 to −0.052)
Fat (g)	0.006 (−0.266 to 0.278)	0.064 (−0.212 to 0.339)	−0.106 (−0.249 to 0.038)
Carbohydrate (g)	0.382 (−0.121 to 0.884)	0.518 (0.009 to 1.027)	0.199 (−0.050 to 0.448)
Cereals (g)	−0.297 (−1.037 to 0.443)	0.060 (−0.683 to 0.803)	−0.135 (−0.827 to 0.557)
Tubers (g)	−0.047 (−0.425 to 0.331)	−0.055 (−0.439 to 0.329)	−0.123 (−0.496 to 0.249)
Vegetables (g)	−0.466 (−1.373 to 0.442)	−0.135 (−1.052 to 0.783)	−0.398 (−1.240 to 0.445)
Fruits (g)	−0.284 (−0.676 to 0.107)	−0.429 (−0.822 to −0.035)	−0.464 (−0.854 to −0.073)
Livestock and poultry (g)	−0.013 (−1.108 to 1.081)	0.196 (−0.910 to 1.302)	−0.405 (−1.131 to 0.322)
Aquatic products (g)	−0.110 (−1.044 to 0.824)	−0.004 (−0.952 to 0.944)	−0.239 (−1.130 to 0.652)
Eggs (g)	−0.199 (−0.483 to 0.084)	−0.163 (−0.450 to 0.124)	−0.237 (−0.505 to 0.031)
Dairy products (g)	0.527 (−0.310 to 1.364)	0.250 (−0.597 to 1.096)	0.077 (−0.736 to 0.890)
Legumes (g)	−0.031 (−0.461 to 0.400)	0.099 (−0.338 to 0.535)	−0.027 (−0.398 to 0.452)
Nuts (g)	−0.169 (−0.289 to −0.049)	−0.171 (−0.294 to −0.049)	−0.183 (−0.304 to −0.062)
Beverages (mL)	1.270 (−0.015 to 2.637)	1.390 (0.087 to 2.683)	1.040 (−0.166 to 2.250)
Snacks (g)	0.461 (0.040 to 0.882)	0.540 (0.113 to 0.966)	0.438 (0.035 to 0.840)

a Model 1 made no adjustment for any covariates. Model 2 was adjusted for age, sex, and socioeconomic status. Model 3 was adjusted for energy intake, age, sex, and socioeconomic status.

b Not available.

Emotional eating behavior also influenced the selections of several food groups. After adjusting for age, sex, and socioeconomic status, higher EESs remained positively associated with a greater tendency to select beverages and snacks, while the selection of fruits and nuts was significantly fewer. After further adjustment for total energy intake, higher EESs were still associated with higher snack selection and fewer selections of fruits and nuts, with no significant associations observed for other food groups (Table 2).

**Figure 4.**
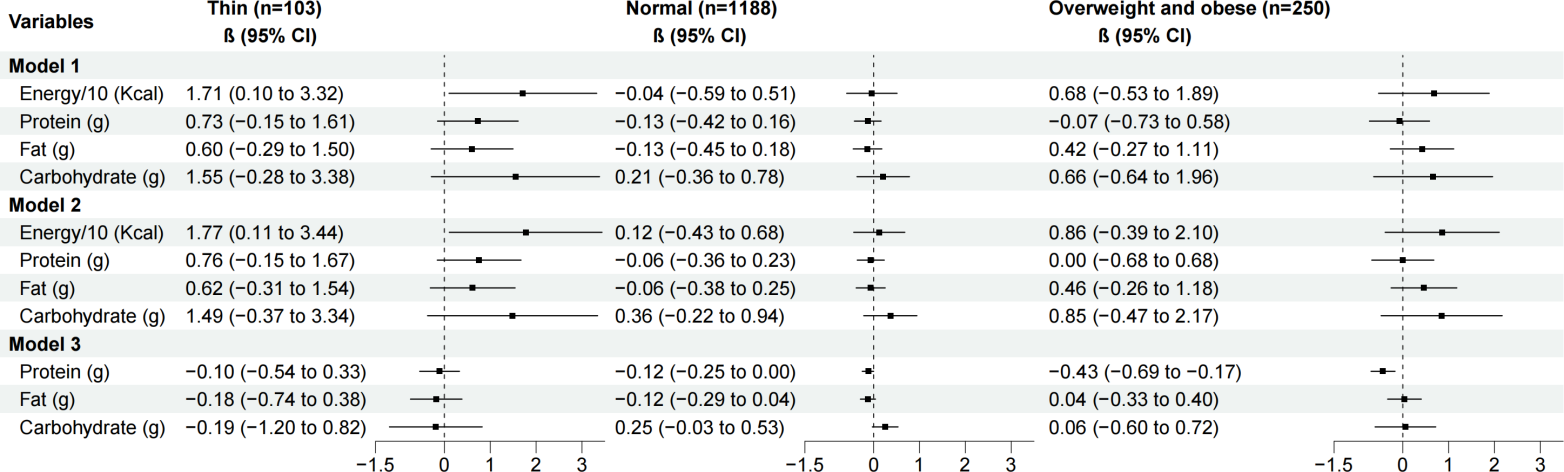
Associations between the emotional eating scale (EES) and intakes of energy and macronutrients stratified by BMI-for-age *z* score (BMI *z* score).

Stratified analysis indicated that higher EESs remained associated with a greater energy intake tendency among the thin population, even after adjusting for age, sex, and socioeconomic status. In the overweight and obese population, a higher EES was associated with a lower tendency to select protein-rich foods, but only after adjusting for age, sex, and total energy intake tendency. In the normal weight population, no significant associations were found between EES and the intake tendencies for energy, protein, carbohydrates, or fat (Figure 4).

Additionally, dynamic scatter plots were used to provide a clear visual representation of how the association between EES and the energy intake tendency levels varied across different BMI *z* scores. However, there is no significant association observed between EES and the intake tendencies of energy, protein, carbohydrates, or fat across different BMI *z* score categories (Multimedia Appendix 4).

We also applied the GDA to investigate the association between dining environment tendencies and nutritional outcomes. In total, 525 (34.1%) participants selected “eat with peers”, while 1016 (65.9%) participants selected “solitary screen eating”, and no significant difference in scenario selection was observed across age, sex, and socioeconomic status. The results presented in Table 3 indicated that participants who wished to eat alone while watching cartoons tended to select 6.9 g more carbohydrates, on average, than those who wished to eat with peers. Even after adjusting for age, sex, and socioeconomic status, those who wished to eat alone while watching cartoons still tended to select 7.7 g more carbohydrates than those who wished to eat with peers. Further adjusting for total energy intake, the difference remained significant, with those who wished to eat alone while watching cartoons tending to select 4.2 g more carbohydrates.

**Table 3.** Effects of dining environment tendencies on intakes of energy, macronutrients, and food groups.

Variables	β (95% CI)		
	Model 1^a^	Model 2	Model 3
Energy (kcal)	4.0 (−1.8 to 9.8)	3.9 (−1.9 to 9.6)	—^b^
Protein (g)	2.3 (−0.7 to 5.4)	2.1 (−1.0 to 5.2)	0.3 (−1.0 to 1.5)
Fat (g)	1.4 (−1.8 to 4.7)	0.9 (−2.3 to 4.2)	−0.9 (−2.6 to 0.8)
Carbohydrate (g)	6.9 (0.9 to 13.0)	7.7 (1.6 to 13.8)	4.2 (1.2 to 7.1)
Cereals (g)	8.6 (−0.3 to 17.5)	10.2 (1.4 to 19.1)	8.1 (−0.2 to 16.3)
Tubers (g)	4.4 (−0.2 to 8.9)	4.8 (0.3 to 9.4)	4.1 (−0.4 to 8.5)
Vegetables (g)	1.3 (−9.7 to 12.2)	1.3 (−9.6 to 12.3)	−1.6 (−11.6 to 8.5)
Fruits (g)	2.2 (−2.5 to 6.9)	1.1 (−3.6 to 5.8)	0.7 (−4.0 to 5.4)
Livestock and poultry (g)	5.8 (−7.4 to 18.9)	3.5 (−9.7 to 16.7)	−3.2 (−11.8 to 5.5)
Aquatic products (g)	1.5 (−9.8 to 12.7)	1.9 (−9.4 to 13.2)	−0.7 (−11.4 to 9.9)
Eggs (g)	4.3 (0.9 to 7.7)	3.8 (0.4 to 7.3)	3.0 (−0.2 to 6.2)
Dairy products (g)	−2.8 (−12.9 to 7.3)	−3.1 (−13.2 to 7.0)	−5.1 (−14.7 to 4.6)
Legumes (g)	−1.3 (−6.5 to 3.9)	−1.0 (−6.2 to 4.2)	−1.8 (−6.8 to 3.3)
Nuts (g)	−1.0 (−2.5 to 0.4)	−1.1 (−2.5 to 0.4)	−1.2 (−2.7 to 0.2)
Beverages (mL)	15.4 (−0.1 to 30.8)	17.7 (2.3 to 33.2)	13.9 (−0.5 to 28.3)
Snacks (g)	2.4 (−2.7 to 7.5)	3.3 (−1.8 to 8.4)	2.2 (−2.6 to 7.0)

a Participants who selected “eating with peers” were used as reference. Model 1 made no adjustment for any covariates. Model 2 was adjusted for age, sex, and socioeconomic status. Model 3 was adjusted for energy intake, age, sex, and socioeconomic status.

b Not available.

Regarding main food groups, participants who wished to eat alone while watching cartoons tended to select 4.3 g more eggs, on average, compared to those who wished to eat with peers, a statistically significant difference. After controlling for age, sex, and socioeconomic status, the difference remained significant, and the former group tended to consume 10.2 g more cereals, 4.8 g more tubers, 3.8 g more eggs, and 17.7 mL more beverages than the latter (Table 3). However, after further adjusting for energy intake, a willingness to eat alone while watching cartoons did not influence the selection of any food group.

## Discussion

This study suggests that the GDA may be a promising approach for simulating dietary choices and assessing eating behavior tendencies, providing insights into developing novel dietary survey methods.

### GDA Could Reflect Some Aspects of Dietary Intakes

In exploratory analysis, we observed moderate associations between the GDA and 24HR for energy, carbohydrate, cholesterol, and certain vitamins, indicating that adolescents’ hypothetical food selections in the GDA may reflect some aspects of their actual intake. However, no significant correlations were found for other nutrients, such as protein, fat, and several micronutrients, suggesting that the GDA may capture only specific dimensions of dietary behavior and not serve as a comprehensive measure of actual intake. This discrepancy may be attributed to the limited food options in the GDA, which cannot fully capture the variety of real-world foods. Despite efforts to include representative foods from different regions of China, a gap remains between the actual food choices of children and adolescents and those offered in the GDA. Similarly, a study using virtual reality technology to simulate dietary choices also found differences between the foods selected in the real world and those chosen in the virtual environment [12].

Additionally, the exploratory study found that the GDA significantly overestimated protein intake, possibly reflecting children’s preferences for protein-rich foods. This finding aligns with the results of the application study, which showed that livestock and poultry meat intake exceeded 200 g, significantly higher than the recommended value in the Dietary Reference Intakes for China [33]. The high proportion of high protein energy ratio distribution may also result from the overestimation. Over the past decades, rapid economic development has significantly increased meat consumption in China [35], and people have become more aware of the importance of protein for the growth and development of children and adolescents, which may have led to a preference for meat among them. Another explanation could be the biased visual presentation or portion sizes of protein-rich foods in the GDA. It may have made these items more appealing, potentially encouraging the selection over other food groups. Excessive meat consumption can increase the risk of obesity [36]; therefore, the meat intake preferences identified in children and adolescents through the GDA should be addressed promptly. Dietary patterns during childhood and adolescence are highly malleable [37], making it crucial to correct unhealthy food preferences at this stage to promote lifelong health. Several other nutrients also showed substantial discrepancies between the GDA and the 24HR, although these differences are significant. Notably, sodium was markedly underestimated in the GDA, likely because the simulated environment includes fewer high sodium processed foods. For vitamin D, the GDA data showed a median (IQR) intake of 3.3 (2.4-4.3) μg, whereas 24HR’s median (IQR) intake was 0 (0-0) since there were only 2 nonzero values. These differences further indicate that the GDA captures preference-driven hypothetical selections rather than actual nutrient intake, underlying the conceptual distinction between the 2 assessment methods.

### Role of the GDA in Uncovering Food Preferences and Eating Behavior Tendencies

In the application analysis, we found the absence of significant differences in GDA-estimated energy selection across weight status groups. This may reflect both developmental and methodological factors. During adolescence, weight status does not necessarily correspond to energy requirements because of large variability in growth and maturation. Besides, the GDA captures hypothetical, preference-based food selection rather than physiological energy needs, allowing adolescents across weight groups to select some high-calorie foods in a virtual setting. Additionally, the limited range of food options in the GDA may further attenuate between-group differences. The primary aim of developing the GDA is to capture dietary behaviors and food preferences among children and adolescents. These aspects are often overlooked and challenging to obtain through traditional dietary surveys. In our application study, we found that the GDA can identify adverse eating behaviors. Individuals with high EESs tended to consume more carbohydrates and less protein, suggesting that they coped with their emotions by selecting high-carbohydrate foods. Regarding food groups, participants with higher EESs showed a greater tendency to choose more beverages and snacks and fewer fruits and nuts, despite median selections for some groups being zero. Previous studies have shown that emotional eating behavior in adolescents was positively associated with higher consumption of high-energy–dense foods, such as cake, ice cream, and beverages [38]. The mean frequency of high-energy–dense food intake was roughly 3 to 4 times higher than that of fruits and vegetables [39]. However, traditional dietary surveys collect data on school- or family-provided meals, potentially masking children’s tendencies for emotional eating. The GDA may help reveal more authentic dietary behavior tendencies, such as emotional eating, by allowing children and adolescents to make food choices without the external constraints typically present in real-life settings—such as parental supervision or school meal policies. In this autonomy-simulated environment, their intrinsic preferences and decision-making patterns—such as a tendency to select high-calorie or high-fat foods—can be more clearly observed. This enables the following targeted psychological counseling and nutrition education, which not only enhances the mental health of children and adolescents but also promotes healthy eating patterns. More interestingly, a positive association between EESs and higher energy selection tendency was observed only in the thin subgroup. This finding suggests that the GDA may be particularly sensitive to identifying latent emotional eating tendencies before they translate into weight gain. Emotional eating behaviors may occur earlier than changes in BMI, especially among thin adolescents. However, this result was preliminary and based on a relatively small sample size of thin groups, which should thereby be interpreted with caution.

In this study, we also identified that the dining environment can impact the nutritional outcomes of children and adolescents [40]. In this study, children and adolescents who selected the solitary screen eating scenario tended to choose more carbohydrates than those in the social eating scenario. Screen time during meals may be the critical influencing factor. A number of studies in Europe [41] and the United States [42] have demonstrated that screen exposure during meals, such as watching television, is significantly associated with obesity in children and adolescents. Therefore, the observed association could be attributable to the simulated screen time, which is known as an adverse eating behavior. Although the observed 7 g difference in daily carbohydrate intake may not be clinically significant in isolation, it may reflect meaningful behavioral tendencies—such as watching screens while eating food—that could cumulatively influence dietary patterns and health outcomes over time, particularly during adolescence. Our finding aligns with previous studies [43], as screen time, such as TV viewing, has been associated with increased consumption of sugar-rich foods, leading to higher carbohydrate intake [44].

### Further Development

In the future, to fully realize the potential of the GDA in dietary assessment, ongoing improvements and upgrades to the GDA are needed. First, to better simulate children’s eating processes and assess dietary intake, the GDA should incorporate a wider variety of foods and regularly update precise nutritional data for each item. Second, the mealtime environment can significantly influence the nutritional and behavioral outcomes of children and adolescents [45,46]. The GDA should therefore enrich meal setting options, such as meal duration and dining location, to maximize its effectiveness in addressing adverse eating behavior tendencies in advance. Finally, gamification has already been successfully applied in developing certain nutrition education games [27,47]. The GDA could also incorporate a food and nutrition education module, making the nutrition education process more engaging and accessible while offering users personalized, practical dietary improvement suggestions.

### Strengths and Limitations

The study used a dual-phase approach, integrating both an exploratory study and an application study. This design not only explored the application of gamification in dietary assessment but also provided insights into the dietary behavior tendencies and food preferences of children and adolescents in a simulated context. However, we acknowledged some limitations. First, this study was conducted solely among populations in 2 Chinese cities, limiting the representativeness of the findings. Future research should involve a larger, multicenter population sample to enhance the generalizability of the results. Second, the exploratory study did not compare the GDA with the weighed record method, which was regarded as the gold standard for dietary assessment but was challenging to implement. The applicability of the findings may also be limited as EES was not specifically validated for our study population of Chinese children and adolescents. Third, this exploratory study included a small sample size (n=30), which may limit the generalizability of the findings. However, the primary aim was to preliminarily assess the consistency between the GDA and 24HR rather than to provide definitive validation or precise estimates. The thin subgroup (n=103) in stratified analysis was also small. This limited sample size reduced the statistical power of the regression models and resulted in wide confidence intervals, suggesting that the observed associations in this subgroup should be interpreted cautiously. Fourth, as participants in the application study were middle school students in an older age range, further research is needed to determine the applicability of the GDA for younger children. Another limitation is that the 24HR did not include weekend days, which might have introduced bias, as dietary patterns can vary between weekdays and weekends. This was due to the on-site data collection schedule during school days. Besides, this study did not investigate user acceptability, which may vary across ages and cultural backgrounds in response to the GDA. In addition, the high rate of exclusion due to failed attention checks (373 out of 2241) suggested potential selection bias since participants who failed attention checks were significantly younger. Besides, in the GDA, the solitary eating scenario involves screen exposure. Therefore, the independent effects of eating alone and screen exposure could not be disentangled. Finally, there are some methodology limitations. As the GDA is designed to capture preference-based dietary behavior tendencies rather than actual intake, several food groups exhibited zero-inflated distributions with median values of zero. These patterns reflect selective hypothetical choices rather than true absence of consumption and may limit the suitability of standard linear regression. There are conceptual and methodological mismatches between the GDA and 24HR. While 24HR aims to quantify 3-day averaged actual nutrient and energy intake, which smooths out daily variations, the GDA is designed to capture 1-time hidden dietary behavior tendencies in a simulated environment. This difference reflects the distinct purposes of the tools and should be considered when interpreting their comparison. Moreover, the gamified nature of the virtual buffet may have encouraged curiosity-driven selections that do not fully reflect habitual dietary behavior, potentially introducing bias unrelated to dietary behavior.

### Conclusions

This study compared the new gamified dietary assessment tool with the interview-led 24HR among children and adolescents for intakes of food groups, nutrients, and energy. While the GDA cannot fully reflect actual food and nutrient intake, it is primarily designed to capture dietary intake tendencies, particularly adverse eating behaviors that may not be easily detected by traditional dietary assessment methods. Despite some discrepancies with 24HR data, the GDA provides valuable insights into patterns of food selection and eating habits tendencies among children and adolescents, which could be hidden in traditional dietary survey methods. The GDA can thus serve as a complementary tool to traditional dietary assessment instruments in the field of dietary survey methods, thereby enhancing the comprehensiveness and accuracy of dietary survey outcomes.

## Supplementary material

10.2196/72387 Multimedia Appendix 1 Cuisines and beverages that are included in the gamified dietary assessment tool (GDA).

10.2196/72387 Multimedia Appendix 2 Distribution of average gamified dietary assessment tool (GDA)–estimated intakes of energy, macronutrients, and food groups with different sociodemographic status.

10.2196/72387 Multimedia Appendix 3 Gamified dietary assessment tool (GDA)–estimated macronutrient energy ratios and their distributions (%).

10.2196/72387 Multimedia Appendix 4 Dynamic scatter plots indicating changing associations between emotional eating scores and intakes of energy and macronutrients with varying BMI *z* scores.

## Abbreviations

24HR: 24-hour dietary recall
AMDR: acceptable macronutrient distribution range
BMI *z* scores: BMI-for-age *z* scores
EES: emotional eating scale
GDA: gamified dietary assessment tool
