# Supplementary material for: Development of a Novel Dietary Assessment Method Using Gamification Concepts: Exploratory and Application Study
Source: JMIR Serious Games. 2026 Mar 13;14:e72387. doi: 10.2196/72387 (PMC12987409; doi:10.2196/72387)
Supplement: Multimedia Appendix 1 [file games-v14-e72387-s001.docx]

## Dishes and beverages contained in GDA

| Meals |  | Dishes and beverages |
| --- | --- | --- |
| Breakfast |  | Stuffed buns, steamed bread, fried eggs, porridge, fried dough sticks, boiled eggs, orange juice, soy milk, Americano coffee, yogurt |
| Lunch and breakfast | Staple food | Rice noodles, hamburgers, steamed bread, rice, pizza, mixed grain rice, noodles, porridge |
|  | Main courses | Boiled prawns, steak, braised beef, braised fish, twice-cooked pork, ham sausage, grilled lamb, smoked meat, fried pork tenderloin, steamed bass, stir-fried minced pork with sour beans, garlic oysters, chicken stewed with mushrooms, fried chicken, fried pork chops, peas with minced meat, tofu pudding, scrambled eggs with tomatoes, stir-fried carrots with black fungus, stir-fried potato with green peppers, stir-fried, kelp and white gourd soup, sauerkraut vermicelli, garlic spinach, seaweed and egg drop soup, vegetable salad |
|  | Snacks and fruits | Spicy sticks, chocolate, biscuits, ice cream, cake, nuts, bread, potato chips, fruit salad, yogurt fruit salad |
|  | Beverages | Tea, orange juice, red wine, mineral water, cola, milk, fruit wine, yogurt, sugar-free cola, Americano coffee, pear drink, iced tea, soy milk, probiotic drink, coconut milk, bubble tea, beer |
