# Supplementary material for: Development of a Novel Dietary Assessment Method Using Gamification Concepts: Exploratory and Application Study
Source: JMIR Serious Games. 2026 Mar 13;14:e72387. doi: 10.2196/72387 (PMC12987409; doi:10.2196/72387)
Supplement: Multimedia Appendix 2 [file games-v14-e72387-s002.docx]

Distribution of average GDA-estimated intakes of energy, macronutrients, and food groups with different socio-demographic status

| Variables | Age | | | Sex | | | Socioeconomic status^a^ | | | | Weight status | | | |
| --- | --- | --- | --- | --- | --- | --- | --- | --- | --- | --- | --- | --- | --- | --- |
|  | 11-14y | 15-18y | *P* | Female | Male | *P* | Low | Medium | High | *P* | Thin | Normal | Overweight and obese | *P* |
| *Energy and Macronutrients* |  |  |  |  |  |  |  |  |  |  |  |  |  |  |
| Energy(kcal)  M (P_25_, P_75_) | 1900.2  (1398.5, 2233.0) | 1860.2  (1419.5, 2209.1) | .32 | 1821.7  (1338.2, 2164.4) | 1906.1  (1439.9, 2249.3) | .003 | 1848.1  (1304.7, 2206.8) | 1870.1  (1421.3, 2208.2) | 1927.7  (1448.0, 2277.2) | .29 | 1846.2  (1580.4, 2268.4) | 1871.4  (1390.8, 2224.7) | 1908.0  (1450.0, 2181.3) | .63 |
| Protein (g)  M (P25, P75) | 98.2  (67.6, 114.6) | 95.7  (70.7, 113.7) | .72 | 93.5  (65.2,112.0) | 98.5  (72.8, 114.6) | .01 | 91.7  (62.8, 112.2) | 97.2  (69.4, 113.5) | 97.8  (74.6, 116.2) | .30 | 98.5  (73.7, 113.6) | 95.7  (67.9, 114.1) | 99.9  (73.6, 113.8) | .44 |
| Fat (g)  M (P_25_, P_75_) | 72.1  (51.3, 94.6) | 75.0  (52.7, 98.4) | .08 | 72.2  (49.1, 92.7) | 75.5  (54.3, 99.3) | .01 | 69.1  (47.9, 93.3) | 74.3  (52.6, 97.4) | 74.5  (54.2, 94.9) | .34 | 74.0  (62.3, 90.9) | 73.7  (51.4, 96.5) | 74.1  (52.9, 99.3) | .69 |
| Carbohydrate (g)  M (P_25_, P_75_) | 211.1  (163.0, 244.4) | 198.7  (153.0, 234.8) | <.001 | 197.7  (154.5, 234.1) | 205.4  (157.8, 242.1) | .04 | 201.5  (153.1, 243.5) | 201.5  (156.5, 237.2) | 207.7  (156.8, 247.6) | .47 | 209.3  (165.7, 241.4) | 201.5  (155.0, 237.6) | 202.4  (158.3, 241.4) | .46 |
| *Main food groups* |  |  |  |  |  |  |  |  |  |  |  |  |  |  |
| Cereals (g)  M (P_25_, P_75_) | 230.7  (172.3, 288.3) | 212.5  (164.1, 270.0) | .02 | 200.4  (150.0, 248.1) | 221.0  (168.8, 282.0) | <.001 | 230.2  (156.2, 284.0) | 210.0  (160.0, 268.7) | 209.9  (156.0, 282.1) | .26 | 216.0  (155.8, 270.1) | 210.0  (158.6, 270.0) | 212.5  (165.7, 270.1) | .91 |
| Tubers (g)  M (P_25_, P_75_) | 58.5  (0, 58.5) | 58.5  (0, 58.5) | .80 | 58.5  (0, 58.5) | 58.5  (0, 58.5) | .21 | 58.5  (0, 58.5) | 58.5  (0, 58.5) | 58.5  (0, 58.5) | .79 | 58.5  (0, 58.5) | 58.5  (0, 58.5) | 58.5  (0, 58.5) | .86 |
| Vegetables (g)  M (P_25_, P_75_) | 209.2  (139.5, 281.5) | 205.9  (135.7, 283.4) | .99 | 189.4  (117.3, 258.4) | 209.1  (135.2, 290.7) | <.001 | 196.3  (119.0, 263.4) | 200.2  (123.9, 279.3) | 212.6  (146.3, 284.2) | .19 | 202.5  (135.5, 269.2) | 199.3  (122.3, 276.2) | 216.2  (135.9, 292.6) | .11 |
| Fruits (g)  M (P_25_, P_75_) | 0  (0, 60.0) | 30.6  (0, 61.2) | .003 | 30.6  (0, 61.2) | 0  (0, 60.0) | <.001 | 0  (0, 60.0) | 30.6  (0, 61.2) | 15.3  (0, 60.0) | .26 | 0  (0, 60.0) | 30.6  (0, 61.2) | 30.6  (0, 60.0) | .15 |
| Livestock and Poultry(g)  M (P_25_, P_75_) | 248.5  (157.6, 330.5) | 262.5  (177.5, 348.6) | .23 | 241.0  (146.9, 324.4) | 255.0  (171.0, 346.8) | .02 | 225.0  (115.5, 321.0) | 252.5  (160.7, 344.3) | 245.5  (166.0, 321.4) | .13 | 259.0  (161.5, 331.0) | 248.5  (151.5, 338.5) | 253.8  (180.0, 346.5) | .68 |
| Aquatic Products (g)  M (P_25_, P_75_) | 180.0  (94.8, 260.0) | 171.0  (96.7, 253.2) | .49 | 163.4  (90, 243.6) | 170.0  (92.9, 253.4) | .08 | 182.9  (90.3, 256.7) | 170.0  (90, 250.0) | 178.2  (94.8, 255.8) | .13 | 182.9  (130.0, 260.0) | 170.0  (90.0, 250.0) | 170.0  (92.9, 250.0) | .20 |
| Eggs (g)  M (P_25_, P_75_) | 39.0  (16.1, 64.1) | 41.0  (25.8, 67.1) | .17 | 39.0  (12.9, 67.1) | 41  (25.8, 67.1) | .09 | 28.1  (12.9, 56.2) | 41.0  (12.9, 67.1) | 39.0  (12.9, 64.8) | .07 | 36.0  (19.4, 66.0) | 39.0  (12.9, 67.1) | 41.0  (28.1, 67.1) | .28 |
| Dairy Products (g)  M (P_25_, P_75_) | 62.7  (6.3, 146.3) | 45.7  (11.8, 149.4) | .82 | 63.3  (16.3, 153.9) | 41.2  (4.5, 136.3) | <.001 | 45.7  (4.5, 136.3) | 45.7  (11.8, 149.4) | 39.8  (4.5, 147.0) | .53 | 50.2  (11.8, 151.7) | 45.7  (11.8, 149.4) | 41.2  (4.5, 131.8) | .12 |
| Legumes (g)  M (P_25_, P_75_) | 0  (0, 90.0) | 0  (0, 58.4) | .02 | 0  (0, 58.4) | 0  (0, 58.4) | .009 | 0  (0, 58.4) | 0  (0, 58.4) | 0  (0, 65.9) | .28 | 0  (0, 58.4) | 0  (0, 58.4) | 0  (0, 58.4) | .91 |
| Nuts (g)  M (P_25_, P_75_) | 0  (0, 0) | 0  (0, 0) | .30 | 0  (0, 0) | 0  (0, 0) | .91 | 0  (0, 0) | 0  (0, 0) | 0  (0, 0) | .50 | 0  (0, 0) | 0  (0, 0) | 0  (0, 0) | .72 |
| Beverages (ml)  M (P_25_, P_75_) | 240.0  (120.0, 360.0) | 240.0  (120.0, 360.0) | .06 | 240.0  (120.0, 360.0) | 240.0  (120.0, 360.0) | .29 | 240.0  (120.0, 360.0) | 240.0  (120.0, 360.0) | 240.0  (120.0, 360.0) | .21 | 240.0  (120.0, 360.0) | 240.0  (120.0, 360.0) | 240.0  (120.0, 360.0) | .33 |
| Snacks (g)  M (P_25_, P_75_) | 78.0  (48.0, 118.8) | 78.0  (48.0, 106.8) | .16 | 78.0  (48.0, 106.8) | 78.0  (48.0, 106.8) | .33 | 69.6  (44.4, 108.0) | 78.0  (48.0, 106.8) | 78.0  (48.0, 107.7) | .82 | 78.0  (48.0, 107.4) | 78.0  (48.0, 106.8) | 78.0  (48.0, 108.0) | .14 |
| ^a^The socioeconomic status variable ranges from 1 to 10, with higher scores indicating a higher socioeconomic status. Scores of 0 to 3 are classified as low, 4 to 7 as medium, and 8 to 10 as high. | | | | | | | | | | | | | | |
