# Supplementary material for: Development of a Novel Dietary Assessment Method Using Gamification Concepts: Exploratory and Application Study
Source: JMIR Serious Games. 2026 Mar 13;14:e72387. doi: 10.2196/72387 (PMC12987409; doi:10.2196/72387)
Supplement: Multimedia Appendix 3 [file games-v14-e72387-s003.docx]

GDA-estimated Macronutrient energy ratios and their distributions (%)

| Variables | Age | | | | Sex | | | Socioeconomics status^a^ | | | | Weight status | | | |
| --- | --- | --- | --- | --- | --- | --- | --- | --- | --- | --- | --- | --- | --- | --- | --- |
|  |  | 11-14y | 15-18y | *P* | Female | Male | *P* | Low | Medium | High | *P* | Thin | Normal | Overweight and obese | *P* |
| Protein energy ratio (%) | M  (P_25_, P_75_) | 20.2  (18.3, 21.8) | 20.4  (18.5, 22.2) | .72 | 20.4  (18.3, 22.1) | 20.3  (18.4, 22.1) | .91 | 20.3  (18.1, 22.2) | 20.4  (18.4, 22.1) | 20.3  (18.5, 21.9) | .98 | 20.2  (18.4, 21.9) | 20.3  (18.3, 22.1) | 20.5  (19.1, 22.0) | .43 |
| Protein energy ratio distribution (%) | <10% | 0.4 | 0.4 | .70 | 0.3 | 0.4 | .99 | 0 | 0.4 | 0.5 | .96 | 1.0 | 0.3 | 0.4 | .18 |
|  | 10%–20% | 44.4 | 42.1 |  | 42.9 | 42.9 |  | 43.7 | 42.6 | 44.3 |  | 46.6 | 43.7 | 37.6 |  |
|  | >20% | 55.2 | 57.5 |  | 56.8 | 56.7 |  | 56.3 | 57.0 | 55.2 |  | 52.5 | 56.0 | 62.0 |  |
| Fat energy ratio (%) | M  (P_25_, P_75_) | 34.8  (30.9, 38.9) | 36.4  (31.8, 42.5) | <.001 | 35.6  (31.3, 40.3) | 35.8  (31.4, 41.9) | .19 | 34.8  (31.1, 41.2) | 36.0  (31.5, 41.1) | 35.1  (31.3, 40.4) | .36 | 35.3  (31.9, 41.3) | 35.7  (31.2, 41.0) | 36.1  (31.9, 41.2) | .92 |
| Fat energy ratio distribution (%) | <20% | 0.8 | 0.7 | .15 | 0.5 | 0.8 | .80 | 0 | 0.8 | 0.5 | .85 | 1.0 | 0.7 | 0.8 | .74 |
|  | 20%–30% | 20.5 | 16.6 |  | 18.1 | 17.8 |  | 20.7 | 17.7 | 17.1 |  | 14.6 | 18.4 | 16.8 |  |
|  | >30% | 78.7 | 82.7 |  | 81.4 | 81.4 |  | 79.3 | 81.4 | 82.4 |  | 84.4 | 80.9 | 82.4 |  |
| Carbohydrate energy ratio (%) | M  (P_25_, P_75_) | 45.3  (41.2, 49.9) | 43.2  (39.2, 48.4) | <.001 | 44.7  (40.6, 49.3) | 43.5  (39.3, 48.5) | .002 | 44.4  (40.0, 50.0) | 43.9  (39.7, 48.8) | 44.2  (39.5, 48.5) | .59 | 44.2  (39.5, 48.5) | 43.9  (39.8, 49.2) | 44.1  (39.7, 48.4) | .91 |
| Carbohydrate energy ratio distribution (%) | <50% | 75.1 | 80.9 | .02 | 77.7 | 79.7 | .37 | 74.8 | 79.2 | 80.0 | .74 | 83.5 | 78.4 | 79.2 | .21 |
|  | 50%–65% | 23.4 | 18.4 |  | 21.6 | 19.2 |  | 24.5 | 19.8 | 19.0 |  | 14.6 | 20.8 | 19.2 |  |
|  | >65% | 1.5 | 0.70 |  | 0.7 | 1.1 |  | 0.7 | 1.0 | 1.0 |  | 1.9 | 0.8 | 1.6 |  |
| ^a^The socioeconomic status variable ranges from 1 to 10, with higher scores indicating a higher socioeconomic status. Scores of 0 to 3 are classified as low, 4 to 7 as medium, and 8 to 10 as high. | | | | | | | | | | | | | | | |
